# A radiomics-based study for differentiating parasellar cavernous hemangiomas from meningiomas

**DOI:** 10.1038/s41598-022-19770-9

**Published:** 2022-09-15

**Authors:** Chunjie Wang, Lidong You, Xiyou Zhang, Yifeng Zhu, Li Zheng, Wangle Huang, Dongmei Guo, Yang Dong

**Affiliations:** 1 Department of Radiology, The Second Hospital of Dalian Medical University, No. 467 Zhongshan Road, Shahekou District, Dalian City, 116023 China; 2 Department of Radiology, Taihe County People’s Hospital, Taihe, China; 3 Department of Radiology, Jining No. 1 People’s Hospital, Jining, China; 4 Department of Radiology, The First Affiliated Hospital of Wenzhou Medical University, Wenzhou, China

**Keywords:** CNS cancer, Clinical trial design

## Abstract

To investigate the value of the radiomic models for differentiating parasellar cavernous hemangiomas from meningiomas and to compare the classification performance with different MR sequences and classifiers. A total of 96 patients with parasellar tumors (40 cavernous hemangiomas and 56 meningiomas) were enrolled in this retrospective multiple-center study. Univariate and multivariate analyses were performed to identify the clinical factors and semantic features of MRI scans. Radiomics features were extracted from five MRI sequences using radiomics software. Three feature selection methods and six classifiers were evaluated in the training cohort to construct favorable radiomic machine-learning classifiers. The performance of different classifiers was evaluated using the AUC and compared to neuroradiologists. The detection rates of T_1_WI, T_2_WI, and CE-T_1_WI for parasellar cavernous hemangiomas and meningiomas were approximately 100%. In contrast, the ADC maps had the detection rate of 18/22 and 19/25, respectively, (AUC, 0.881) with 2.25 cm as the critical value diameter. Radiomics models with the SVM and KNN classifiers based on T_2_WI and ADC maps had favorable predictive performances (AUC > 0.90 and F-score value > 0.80). These models outperformed MRI model (AUC 0.805) and neuroradiologists (AUC, 0.756 and 0.545, respectively). Radiomic models based on T_2_WI and ADC and combined with SVM and KNN classifiers have the potential to be a viable method for differentiating parasellar hemangiomas from meningiomas. T_2_WI is more universally applicable than ADC values due to its higher detection rate for parasellar tumors.

## Introduction

Parasellar cavernous hemangiomas (CHs) are relatively rare intracranial-extraaxial vascular malformation with unknown etiology, accounting for 2–3% of all cavernous sinus tumor^1^. In recent years, advances in neuroradiology techniques improve the ability to detect it^2–4^. Dural cavernous angiomas occurred in parasellar cavernous sinus often share a similar appearance with parasellar meningiomas on conventional MR imaging (MRI) and exhibit a dural tail sign on enhanced-T_1_weighted (T_1_WI) imaging occasionally^5^. They were misdiagnosed as meningiomas commonly^6–9^, and the misdiagnosis rate was as high as 66.7–87.5%^1,10^.

Althouth parasellar CHs were benign, clinical symptoms such as headache and cranial nerve deficits may arise due to progressive tumor growth and mass effect^11^. The management of parasellar CHs remains a challenge for neurosurgeons due to the complex neurovascular structures of the cavernous sinus. The incidence of uncontrollable and massive hemorrhage during surgery and neurovascular function injury was high and even death^12^. Stereotactic radiosurgery (SRS) could alleviate symptoms and effectively reduce surgical complication^12^, attaining long-term CHs control^13,14^. However, SRS increases the risk of adhesion between meningiomas and surrounding tissues, which is not the preferred method for meningiomas. Surgical resection is considered to be an effective strategy for the treatment of parasellar meningiomas^15^, and SRS is an adjuvant treatment for residual or recurrent meningiomas after surgery^5,16,17^. Consequently, accurate preoperative diagnosis for parasellar CHs and meningiomas is crucial for individualized treatment decisions.

In recent years, advanced functional imaging features were explored to provide information for improving diagnostic accuracy, including a description of compactness of tumor cell arrangement, cerebral blood perfusion, and vascular proliferation characteristics. Pathologically, CHs can be classified as type A, B and C^18^. Type A was sponge-like with intact pseudocapsule; type B was mulberry-like with the pseudocapsuel incomplete or absent; and type C was composed of both mulberry-like composition and sponge-lied composition. Parasellar meningiomas are mostly meningothelial subtype^19^. They are obvious enhancement and hyperperfusion, with a significantly lower minimum apparent diffusion coefficient (min ADC) compared to parasellar CHs^20^. These provide valuable information for the identification. However, sometimes its clinical application is limited due to the following reasons: (1) the gradual “filling” features on dynamic contrast-enhanced MRI (DCE-MRI) help in the diagnosis of cavernous hemangioma, which was different from meningomas. However, type A CH, accounting for about 40% of all parasellar CHs^21^, is composed of thin-walled large lumen sinusoids with scanty intervening connective tissue. It shows marked homogeneous enhancement than type B and C^18,22^, which is similar to meningiomas; (2) identification by perfusion status is typically incomplete^23^. Type B cavernous hemangioma contains ample solid parenchyma and well-formed vasculature and connective tissue. It has high CBF values and is easily misdiagnosed as meningiomas^20,22^; (3) poor imaging effect on diffusion-weighted imaging (DWI) of parasellar lesions was inevitably, due to the low signal-to-noise ratio and magnetic susceptibility artifacts caused by skull base bone and nasal containing gas; (4) although DCE-MRI has certain value in differential diagnosis, it is inevitable to inject exogenous contrast agents. Which limits its use in specific populations of pregnant women^18,20,24^. The previous reports showed that parasellar CHs and meningoma were both the most frequently diagnosed parasellar disease during pregnancy^25^. Therefore, the exploration based on conventional MR without contrast agents is more expected.

Radiomics has become an attractive technique in recent years. It is a powerful tool for constructing decision-support models based on conventional or functional imaging for extracting large amounts of image features and quantitative data analysis^26^. However, to our knowledge, its application in differentiating parasellar CHs from meningioma has not been reported^27–30^. The present study extracted a large panel of radiomics features from T1-weighted images (T_1_WI), T2-weighted images (T_2_WI), contrast-enhanced T1-weighted images (CE-T_1_WI), diffusion-weighted imaging (DWI), and apparent diffusion coefficient (ADC) imaging data involving 96 patients with parasellar CHs and meningiomas. This study aimed to construct an MRI-based radiomics model as a noninvasive preoperative prediction method to facilitate the differentiation of parasellar CHs from meningiomas.

## Materials and methods

### Patients

**Figure 1:**
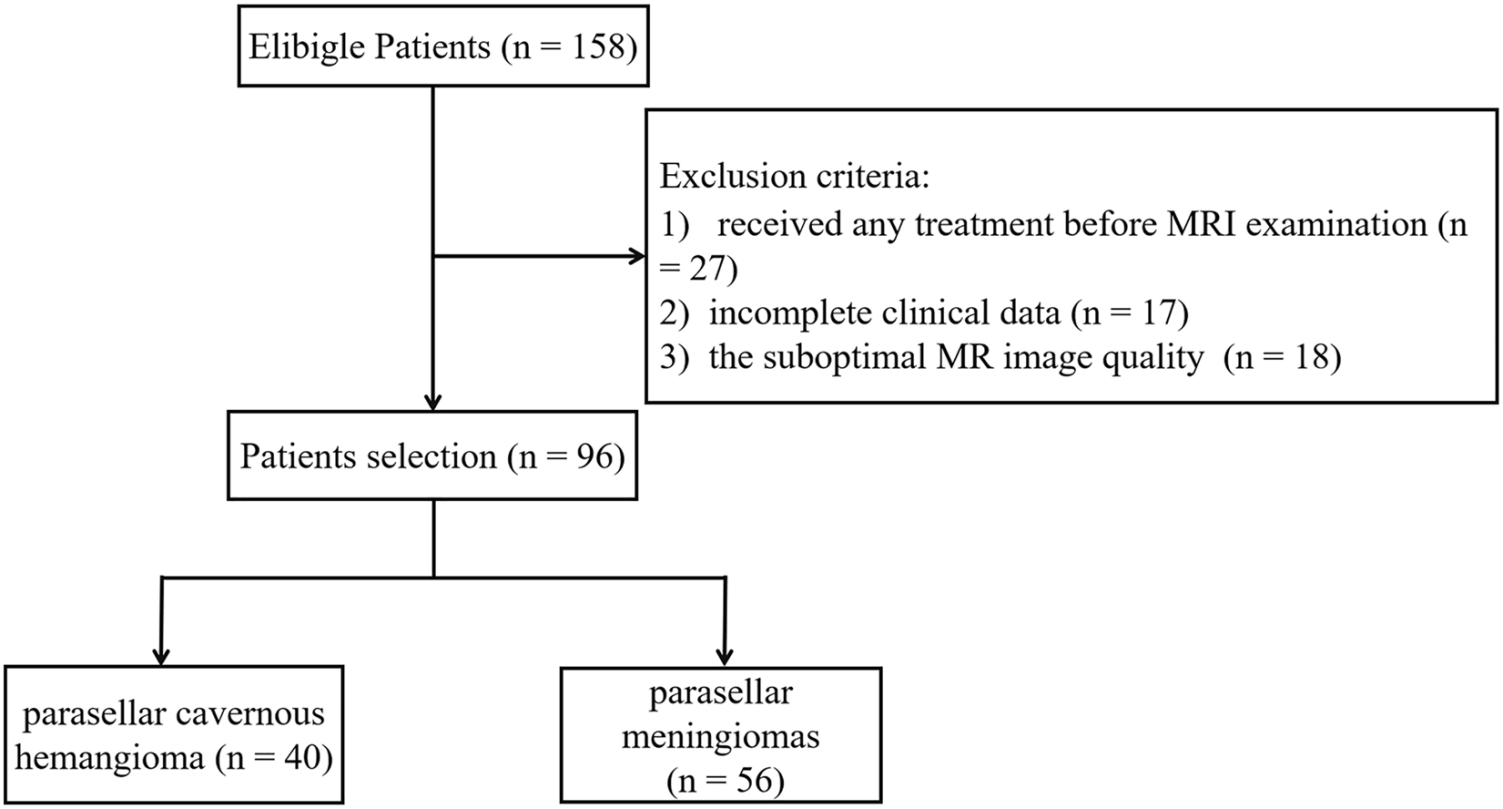
Flowchart for patient selection.

Radiological and clinical databases of 96 cases of parasellar CHs and meningiomas from Second Hospital of Dalian Medical University, Jining NO. 1 People's Hospital, and First Affiliated Hospital of Wenzhou Medical University were retrospectively reviewed between January 2010 and September 2019. This retrospective study was approved by the ethics review board of Second Hospital of Dalian Medical University, Jining NO. 1 People's Hospital, and First Affiliated Hospital of Wenzhou Medical University. The requirement for informed consent was waived by our Review Board owing to the retrospective nature of the current study. The methods in the current study were performed in accordance with the relevant guidelines and regulations. Inclusion criteria included the following: (1) patients pathologically confirmed and/or clinically diagnosed with parasellar cavernous hemangioma or meningioma; (2) preoperative multi-parametric MRI scans including T_1_WI, T_2_WI, CE-T_1_WI, DWI, and ADC data were acquired; and (3) patients with no treatment history before magnetic resonance examination. Patients were excluded if (1) clinical data were incomplete; (2) they received any treatment before the MRI examination, and (3) MR image quality was suboptimal. As a result, 40 cases of parasellar CHs and 56 cases of parasellar meningiomas were included in the study. The flowchart for patient selection is presented in Fig. 1.

### MR image acquisition and data management

MR examinations were performed in 37 and 59 patients using 1.5T (HDXT, GE Healthcare, USA) and 3.0T (Siemens, Verio, Germany) MR scanners, respectively. The MR scan parameters are summarized in Table 1. CE-T_1_WI was acquired after administration of 0.1 mmol/kg of gadolinium-based contrast material (Gadovist; Bayer, Leverkusen, Germany). Diffusion-weighted images were transferred to a post-processing workstation to obtain ADC maps. MR data for T_1_WI, T_2_WI, and CE-T_1_WI were acquired for all patients. DWI was obtained for 27 patients with cavernous hemangiomas and 32 patients with meningiomas. ADC maps were obtained for 22 patients with cavernous hemangiomas and 25 patients with meningiomas. All T_1_WI, T_2_WI, DWI, ADC, and CE-T_1_WI data were selected for texture analysis.

**Table 1:** MRI protocol.

Sequences	TR (ms)	TE (ms)	NEX	Slice Thickness (mm)	FOV (mm)	Matrix
SE-T_1_WI	1750–2500	9–25	2–4	3–5	24 × 24	256 × 256
FSE-T_2_WI	4000–4500	90–120	2	3–5	24 × 24	256 × 256
DWI	4500–6400	70–80	2	3–5	24 × 24	256 × 256

*SE* spin echo, *FSE* fast spin echo, *TR* repetition time, *TE* echo time, *NEX* number of excitations, *FOV* field of view, *DWI* diffusion-weighted imaging.

### Tumor segmentation

The radcloud platform (Huiying Medical Technology Beijing Co., Ltd, https://mics.huiyihuiying.com/#/) was used to manage the imaging and clinical data and to perform subsequent radiomics statistical analysis. To minimize the MRI intensity variations, we normalized the intensity of the image using the following formula:

$$f(x) = \frac{{s(x - \mu_{x})}}{{\delta_{x}}}$$

*x* indicates the original intensity; *f*(*x*) indicates the normalized intensity; *μ* refers to the mean value; *σ* indicates the variance; *s* is an optional scaling, by default, it is set to 1^31^.

**Figure 2:**
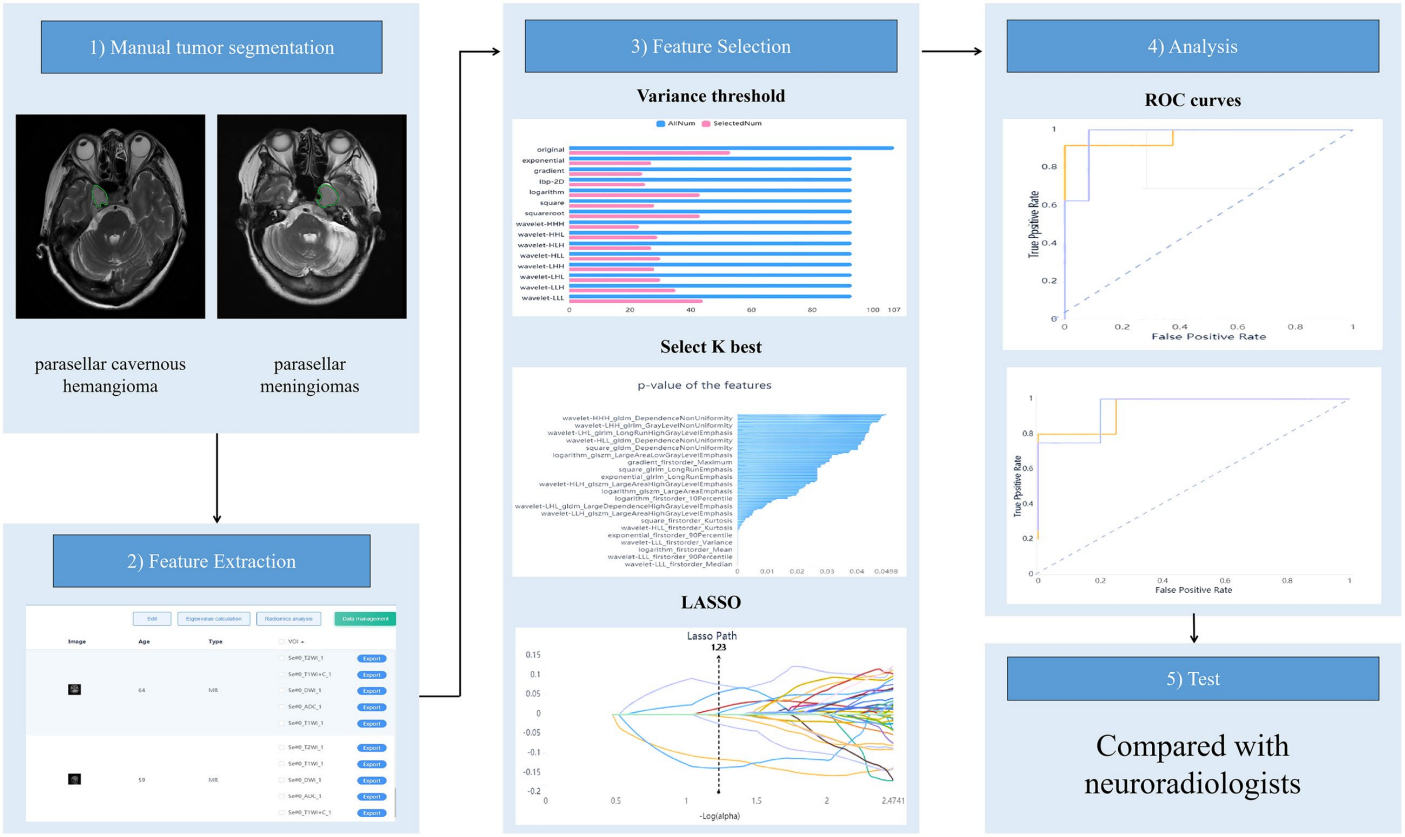
Radiomics workflow.

All lesions in the training set were manually delineated by a junior radiologist on contiguous T_2_WI slices and then copied to the corresponding T_1_WI, CE-T_1_WI, DWI, and ADC maps for each slice. The first and last image layers were excluded to reduce the partial volume effect in all of the following series. The volume of interest (VOI) was manually adjusted to avoid interference from magnetic sensitivity artifacts. A senior radiologist with 10 years of experience reviewed all contour lines and decided on the tumor boundaries when no consensus was reached. Next, the computer automatically generated a three-dimensional VOI. Both radiologists were double-blinded to both clinical and pathological information. Figure 2 depicts a schematic of the radiomics workflow.

### Feature extraction and selection

A total of 1409 quantitative imaging features were extracted from MR images using the Radcloud platform^32^. All of these features were classified into four categories^26,33^. (1) first order statistic: these features quantitatively described the intensity distribution of voxels in MR images, but did not involve the spatial arrangement of voxels; (2) shape-based: these features reflected the shape of the depicted region; (3) texture: texture analysis quantified the variation of features within gray levels and described the statistical information related to the spatial distribution of gray levels or voxel intensities. This analysis was generally performed by second- or higher-order statistical methods that quantified the heterogeneity within the lesion. These features included gray level run length matrix, (GLRLM), gray level co-occurrence matrix (GLCM), and gray level size zone matrix, (GLSZM); (4) high order features: high order features were obtained using statistical methods after filtering the images. They included Laplacian of Gaussian, wavele, square, square root, and logarithm.

**Figure 3:**
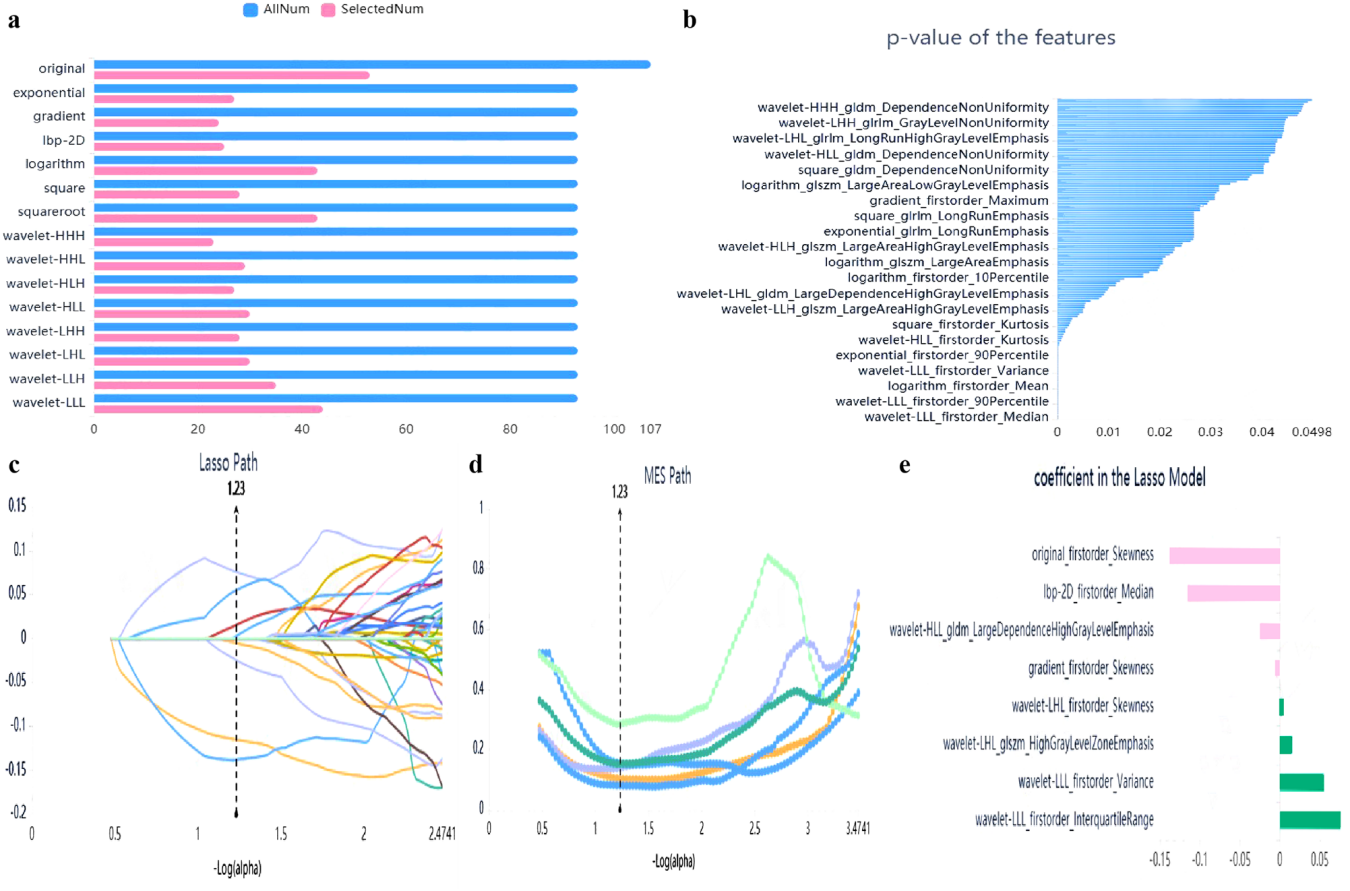
Dimension reduction analysis and feature selection for T_2_WI. (**a**) Variance threshold method was used to select 486 features from 1409 radiomics features (variance threshold = 0.8); (**b**) 145 feat ures were retained using select K best (*P* value < 0.05); (**c**–**e**) 145 features were retained using LASSO algorithm method. Eight eigenvalues were retained.

In order to avoid over-fitting and improve the generalization ability of the model, variance threshold, select K best, and LASSO algorithm were used to select the optimal features (Fig. 3). A variance threshold of 0.8 was used in the variance threshold method to remove variance eigenvalues smaller than 0.8. The select K best was chosen to remove features without a statistically significant difference (*p* > 0.05). For the LASSO model, the L1 regularizer was used as the cost function with a cross-validation error value of 5 and a maximum number of iterations of 1000. The LASSO algorithm was used to find the best alpha in each sequence, calculate the coefficients, and obtain the most relevant features.

### Model training and validation

The present study constructed radiomics-based models using KNN, SVM, LR, RF, XGBoost, and DT classifiers. The radiomic features after a three-dimensional dimensionality reduction were used as the dataset. Then, 80% of the datasets were randomly selected to build the training set and the remaining 20% were used as the validation set to evaluate the accuracy of the models.

### Neuroradiologist evaluation

Subsequently, two neuroradiologists (with 5 and 10 years of experience, respectively) made a diagnosis based on the characteristics of parasellar cavernous hemangiomas and meningiomas in conventional MR images (T_1_WI, T_2_WI, and CE-T_1_WI), including size, signal intensity on T_2_WI and DWI (hyperintensity, isointensity, hypointensity), morphology (roundish, irregular and spindle), the spatial relationship with the peripheral blood vessels (encapsulation, compression, close to, separation), and enhancement characteristics (homogeneous and heterogeneous). Signal intensities were recorded according to the Elster scoring criteria^34^. The lesion diameters detected by ADC maps, T_2_WI, and other sequences were recorded to compare the recognition rate of each sequences. The maximum values were taken as the focus size in this study. The two neuroradiologists were blinded to the clinical and pathology data of specific cases, but knew the patients were parasellar CHs or meningoma.

### Statistical analysis

The present study compared and analyzed the area under the receiver operating curve (ROC) curve with 95% confidence interval (CI), sensitivity, specificity, and accuracy of each classifier based on the results of different MR sequence tests. Model stability was evaluated using the F-score value. The larger the F-score value, the better the stability of the model. The lesion detection rate on different MR images was also analyzed, and the relationship between lesion diameter and the detection rate on ADC maps was statistically evaluated using the SPSS 22.0 software (SPSS, Inc, Chicago, IL). Long-distance cut-off values for the Yoden index findings were obtained based on the data sensitivity and specificity. The performance of the two neuroradiologists was evaluated using ROC curve analysis and compared to the performance of the final radiomics models.

## Results

### Clinical and MRI characteristics

**Figure 4:**
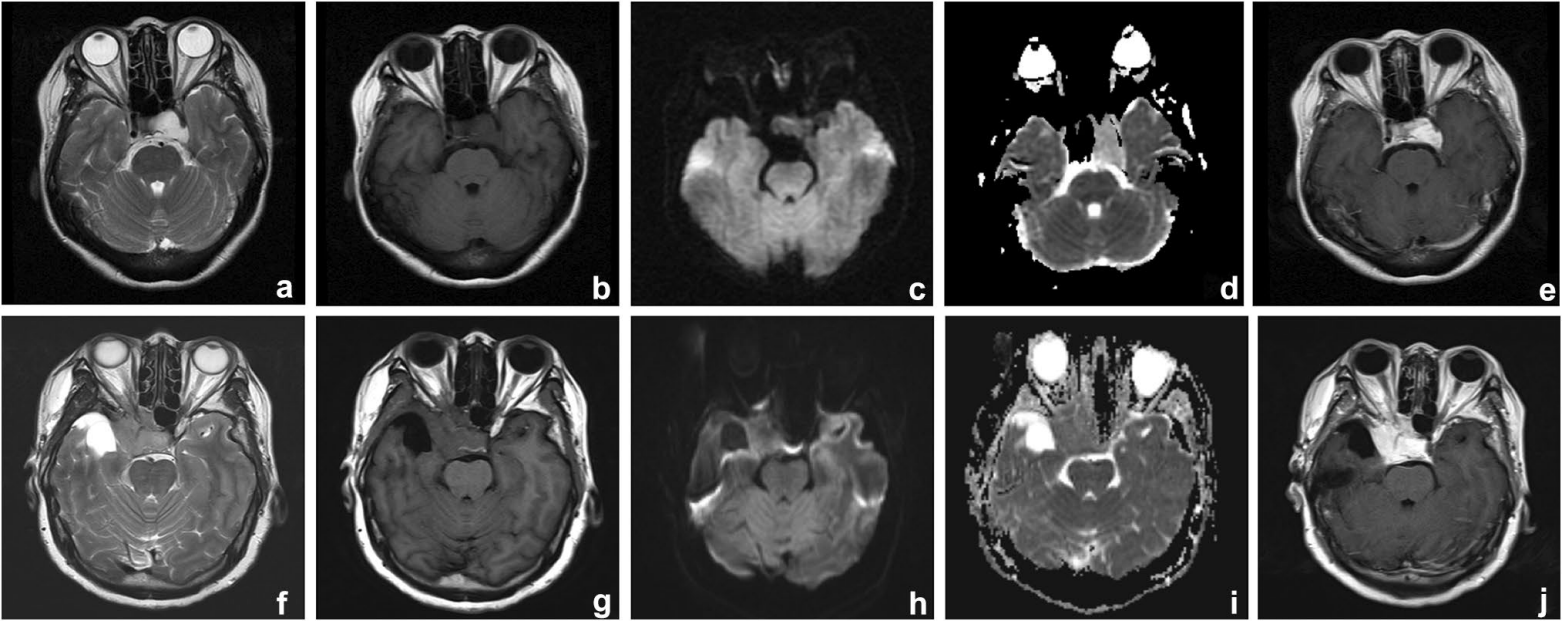
(**a**–**e**) Images of parasellar cavernous hemangioma in a 54-year-old woman. (**f**–**j**) Images of parasellar meningioma in a 58-year-old woman. MRI protocol included (**a**, **f**) axial T_2_-weighted images, (**b**, **g**), axial T_1_-weighted images, (**c**, **h**) diffusion-weighted images, (**d**, **i**) apparent diffusion coefficient maps, and (**e**, **j**) contrast-enhanced T_1_-weighted images. Cavernous hemangioma exhibited hyperintensity on T_2_-weighted images, hypointensity on T_1_-weighted images, DWI, and ADC map, and CE-T_1_WI showed homogeneous enhancement. Meningioma exhibited slightly hyperintensity on T_2_-weighted images, slightly hypointensity on T_1_-weighted images, DWI, and ADC map, and CE-T_1_WI showed homogeneous enhancement.

**Figure 5:**
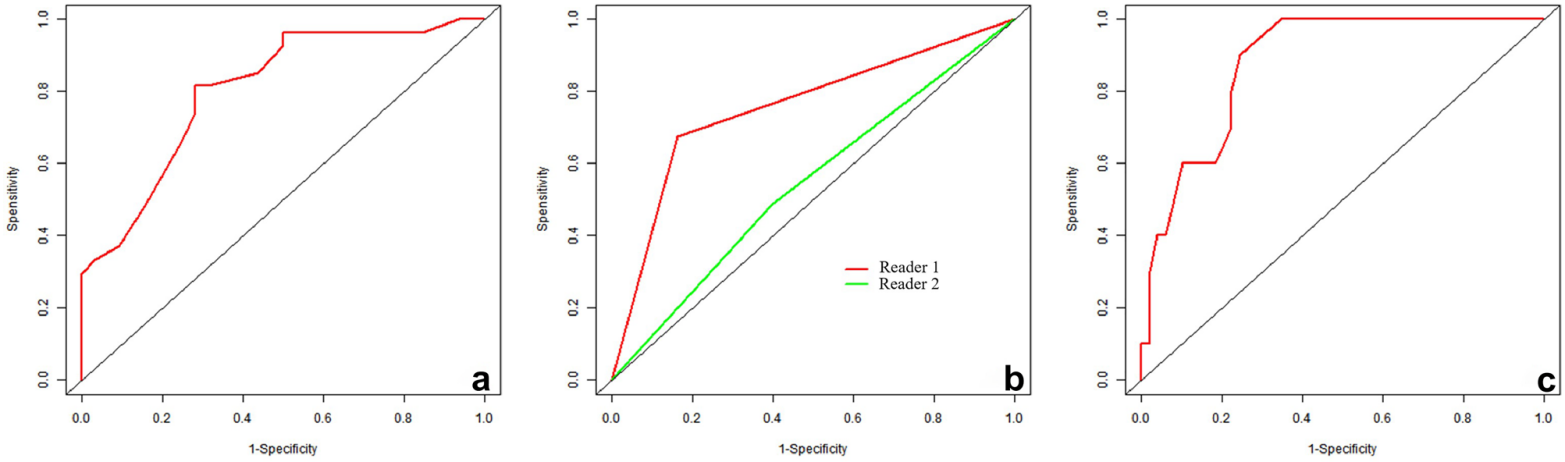
Receiver operating characteristic (ROC) curves of MRI model (**a**, AUC = 0.805), diagnostic efficiency of two neuroradiologists (**b**, the AUC of Reader 1 = 0.756, the AUC of Reader 2 = 0.545), and ADC map detection rate (**c**, AUC = 0.881).

The baseline clinical factors and the semantic image analysis of 96 patients are reported in Table 2. In univariate analyses, signal intensity on T_2_WI and DWI, morphology, the enhancement pattern and the spatial relationship with the peripheral blood vessels showed statistical significance between cavernous hemangiomas and meningiomas (χ^2^ = 35.521, *P* = 0.000; χ^2^ = 9.731, *P* = 0.008, χ^2^ = 7.636, *P* = 0.022, and χ^2^ = 13.253, *P* = 0.004, respectively). No significant differences in age, sex, and size were observed between cavernous hemangiomas and meningiomas (*P* = 0.186, *P* = 0.420 and *P* = 0.212, respectively). In multivariate analyses, signal intensity on T_2_WI , signal intensity on DWI , and the enhancement pattern were demonstrated as independent predictors of semantic features of MRI scans (Table 2). All lesions were detectable on conventional MR images (Fig. 4). The detection rate was 18/22 for cavernous hemangiomas and 19/25 for meningiomas on ADC maps. The area under the curve (AUC) for the detection rate was 0.881 (95% CI 0.790–0.972), with an accuracy, sensitivity, and specificity of 74.2%, 67.3%, and 100%, respectively (Fig. 5c). The mean diameter was approximately 2.74 ± 0.98 cm, with a critical value of 2.25 cm for the diameter on ADC maps.

**Table 2:** Baseline characteristics and semantic image analysis of the population study.

Characteristic	All Patients (*n* = 96)	Cavernous hemangioma (*n* = 40)	Meningiomas (*n* = 56)	Univariate analysis		Multivariate analysis	
				Statistics	*P* Value	Odds Ratio*	*P* Value
Age (y)	58.14 ± 12.02	56.20 ± 14.05	59.52 ± 10.10	7.708	0.186	NA*	NA*
	(23–86)	(23–86)	(26–80)				
**Sex**				0.649	0.420	NA*	NA*
Men	27/96 (0.28)	13/40 (0.33)	14/56 (0.25)				
Wemen	69/96 (0.72)	27/40 (0.67)	42/56 (0.75)				
Size (cm)	3.20 ± 1.09 (1.57–6.80)	3.11 ± 1.19 (1.70–6.80)	3.08 ± 0.96 (1.57–5.6)	9.987	0.212	NA*	NA*
**Signal intensity on T**_**2**_**WI**				35.521	0.000		
Hyperintensity	40/96 (0.42)	24/40 (0.60)	16/56 (0.29)				
Isointensity	39/96 (0.40)	11/40 (0.28)	28/56 (0.50)			3.488 (0.632–19.251)	0.152
Hypointensity	17/96 (0.18)	5/40 (0.12)	12/56 (0.21)			18.194 (1.177–281.334)	0.038
**Signal intensity on DWI**				9.731	0.008		
Hyperintense	21/59 (0.36)	4/27 (0.15)	17/32 (0.53)				
Isointensity	25/59 (0.42)	16/27 (0.59)	9/32 (0.28)			0.060 (0.009–0.404)	0.004
Hypointensity	13/59 (0.22)	7/27 (0.26)	6/32 (0.19)			0.147 (0.019–1.113)	0.063
**Morphology**				7.636	0.022	NA*	NA*
Roundish	41/96 (0.43)	11/40 (0.28)	30/56 (0.54)				
Irregular	48/96 (0.50)	24/40 (0.60)	24/56 (0.43)				
Spindle	7/96 (0.07)	5/40 (0.12)	2/56 (0.03)				
**The spatial relationship with the peripheral blood vessels**				13.253	0.004	NA*	NA*
Encapsulation	55/96 (0.57)	31/40 (0.78)	24/56 (0.43)				
Compression	14/96 (0.15)	5/40 (0.12)	9/56 (0.16)				
Close to	17/96 (0.18)	3/40 (0.08)	14/56 (0.25)				
Separation	10/96 (0.10)	1/40 (0.02)	9/56 (0.16)				
**Enhancement pattern**				11.497	0.001		
Homogeneous	62/96 (0.65)	18/40 (0.45)	44/56 (0.79)				
Heterogeneous	34/96 (0.35)	22/40 (0.55)	12/56 (0.21)			4.979 (1.060–23.389)	0.042

*NA* not analyzed, *DWI* diffusion-weighted imaging.

The AUC of the MRI model (0.805) were lower than those of the radiomics (Fig. 5a). The AUCs for the two neuroradiologists were 0.756 (95% CI 0.654–0.858) for reader 1 (Fig. 5b) and 0.545 (95% CI 0.430–0.659) for reader 2 (Fig. 5b). When comparing diagnostic performance, the radiomics classifier had a significantly higher AUCs than the two neuroradiologists (*P* < 0.001).

### Model assessment

After three-dimensionality reductions, eight out of 1409 features were selected based on T_2_WI (Table 3). Features based on other sequences are listed in Supplementary Tables S1–S4.

**Table 3:** Description of selected radiomic features with their associated feature group and filter based on T_2_WI.

Radiomic feature	Radiomic class	Filter
Median	firstorder	Lbp-2D
Interquartile range	firstorder	Wavelet-LLL
Variance	firstorder	Wavelet-LLL
Skewness	firstorder	Original
Skewness	firstorder	Gradient
High gray level zone emphasis	glszm	Wavelet-LHL
Large dependence high gray level emphasis	gldm	Wavelet-HLL
Skewness	firstorder	Wavelet-LHL

*GLDM* gray-level dependence matrix, *GLSZM* gray-level size zone matrix.

**Figure 6:**
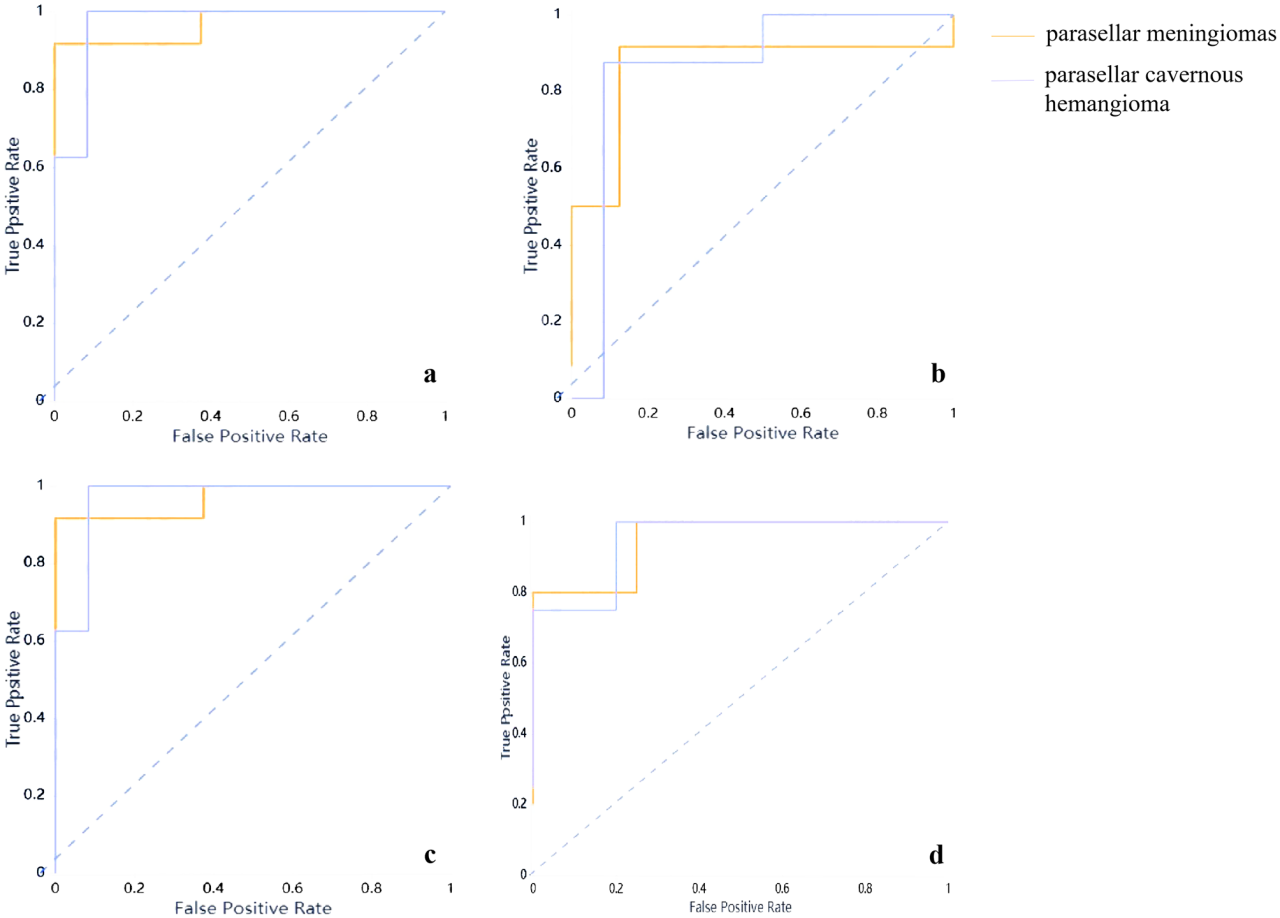
ROC curves for the optimal classifier. (**a**) ROC curve for KNN model based on T_2_WI with AUC = 0.93; (**b**) ROC curve for SVM model based on T_2_WI with AUC = 0.88; (**c**) ROC curve for KNN model based on ADC maps with AUC = 0.83; (**d**) ROC curve for SVM model based on ADC maps with AUC = 0.81.

The diagnostic performance of the prediction models is summarized in Tables 4 and 5. After removing all over-fitting results for recognizable lesions, the T_2_WI-based radiomics model with KNN and SVM classifiers was more effective in identifying parasellar cavernous hemangiomas from meningiomas (Fig. 6).

**Table 4:** Performance of KNN classifier radiomics models in differentiating parasellar cavernous hemangiomas from meningiomas in the validation set.

MRI sequence	Category	AUC	95% CI	Sensitivity	Specificity	F-score
T_2_WI	Meningiomas	0.93	0.78–1.00	0.92	0.88	0.9
	Cavernous hemangioma	0.93	0.78–1.00	0.88	0.92	0.88
ADC	Meningiomas	0.93	0.75–1.00	0.88	1	0.89
	Cavernous hemangioma	0.93	0.75–1.00	1	0.88	0.89
CE-T_1_WI	Meningiomas	0.92	0.69–1.00	0.82	0.71	0.82
	Cavernous hemangioma	0.92	0.69–1.00	0.71	0.82	0.71
DWI	Meningiomas	0.79	0.56–1.00	0.5	1	0.67
	Cavernous hemangioma	0.79	0.56–1.00	1	0.5	0.80
T_1_WI	Meningiomas	0.75	0.55–0.94	0.83	0.75	0.83
	Cavernous hemangioma	0.75	0.55–0.94	0.75	0.83	0.75

*MRI* magnetic resonance imaging, *T*_*1*_*WI* T_1_-weighted images, *T*_*2*_*WI* T_2_-weighted images, *DWI* diffusion-weighted images, *CE-T*_*1*_*WI* contrast-enhanced T_1_-weighted images, *AUC* areas under the ROC curves, *95% CI* 95% confidence interval.

**Table 5:** Performance of SVM classifier radiomics models in differentiating parasellar cavernous hemangiomas from meningiomas in the validation set.

MRI sequence	Category	AUC	95% CI	Sensitivity	Specificity	F-score
T_2_WI	Meningiomas	0.87	0.71–1.00	0.92	0.88	0.92
	Cavernous hemangioma	0.87	0.71–1.00	0.88	0.92	0.88
ADC	Meningiomas	0.95	0.77–1.00	0.88	1	0.89
	Cavernous hemangioma	0.95	0.77–1.00	1	0.88	0.89
CE-T_1_WI	Meningiomas	0.91	0.73–1.00	1	0.71	0.92
	Cavernous hemangioma	0.91	0.73–1.00	0.71	1	0.83
DWI	Meningiomas	0.94	0.71–1.00	0.67	1	0.80
	Cavernous hemangioma	0.94	0.71–1.00	1	0.67	0.86
T_1_WI	Meningiomas	0.73	0.52–0.94	0.75	0.75	0.78
	Cavernous hemangioma	0.73	0.52–0.94	0.75	0.75	0.71

*MRI* magnetic resonance imaging, *T*_*1*_*WI* T_1_-weighted images, *T*_*2*_*WI* T_2_-weighted images, *DWI* diffusion-weighted images, *CE-T*_*1*_*WI* contrast-enhanced T_1_-weighted images, *AUC* areas under the ROC curves, *95% CI* 95% confidence interval.

## Discussion

The present study established an accurate classifier to distinguish parasellar cavernous hemangiomas from meningiomas by integrating a large panel of radiomic features. An efficient classifier was obtained by comparing five MRI sequences from 1.5 T and 3.0 T MR scanners at three medical imaging centers, bolstering its generalizability. Through radiomic and artificial evaluation, T_2_WI and DWI sequences were of great value in the differentiation of parasellar CHs and meningoma, outperforming the enhanced-T_1_WI. And T_2_WI is more universal applicable for its less artifacts and higher detection rate of parasellar lesion. MRI-based radiomic models would be a potential method for differentiating parasellar CHs from meningomas.

In this study, imaging characteristics of parasellar CHs and meningomas were analyzed. It was found that the signal intensity on DWI and T_2_WI, and the enhancement mode in contrast-enhanced MR imaging had advantages in the differention of them. The previous study reported that the facilitated diffusion on DWI could differentiate parasellar CHs from other lesions^35^. In this study, ADC sequences had a good practical value in constructing radiomics models. However, the detection rate of parasellar CHs and meningoma in DWI and ADC maps was about 78.7% (37/47), with a cut-off diameter of 2.25 cm. Which affects the clinical application of this technology. Well, the detection rate of T_2_WI, T_1_WI, and CE-T_1_WI was 100%, which was more conducive to the establishment of radiomics models. This study proposes for the first time that the signal intensity on T_2_WI is also significant for the identification. It was characterized by a high signal-to-noise ratio and homogeneity^27,36^. The radiomics model constructed based on T_2_WI had a high diagnostic accuracy and stability in distinguishing parasellar hemangiomas and meningiomas, which provides a methodological basis for diagnosis when advanced functional and enhanced MR are difficult to carry out. The progressive contrast “filling in” in the tumors can aid in differentiating between them, which was reported in the previous studies and suggested the diagnosis of cavernous hemangiomas^27,37^. However, contrary to our general view, the accuracy of the radiomics model based on CE-T_1_WI was low than T_2_WI and ADC, although it was improved in different ways. This might be influenced by different types of cavernous hemangiomas and meningiomas^22,37,38^, which is worthy of further study.

Radiomics can provide additional metabolic and biological information in addition to the traditional MRI metrics. Gray contrast, uniformity, depth, and texture roughness have been used to study tumor grading, prediction of genomic information, and differentiation of lesion and non-lesion images^39–41^. The present study found that higher-order features could better reflect the degree of tumor heterogeneity and texture information. A GLSZM can quantify gray-level zones in an image to reflect tumor heterogeneity at a local scale. The coefficient of High Gray-Level Zone Emphasis was the largest, which measured the distribution of the higher gray-level values. Larger values indicated a larger proportion of high gray-level values and size zones in the image^42^. Tumor heterogeneity usually reflected the gray contrast variation of the image. Therefore, the GLSZM was more sensitive in distinguishing parasellar cavernous hemangiomas from meningiomas.

Different classifier algorithms may lead to different results. The present results suggested that the radiomics models combined with SVM and KNN classifiers had better diagnostic performance in distinguishing between parasellar cavernous hemangiomas and meningiomas. SVM has been proposed by Cortes et al. in 1995 as a binary classifier based on supervised learning^43,44^. The critical concept of SVM involves the use of a hyperplane to define decision boundaries to separate different classes of data points. This technique finds support vectors with a high discrimination and maximizes the interval between classes. It has good adaptability and discrimination ability. The K-nearest neighbor (KNN) method is mostly used for image classification. This object classification is based on the distance between its neighbors and is mainly used to solve regression and classification problems. By selecting the KNN points of a sample when the nearest neighbors belong to a certain category, the sample is determined to belong to that category. Several previous studies have demonstrated KNN's excellent and stable performance using different datasets, which was similar to the present result^45–47^. Consistent with our study, other classifiers also suffer from over-fitting. This is manifested by the fact that the training set is too accurate, while the validation set cannot achieve the expected ideal results. In addition, there are too many feature dimensions, parameters, and noise, which lead to a too-perfect prediction of the fitted function in the training set. However, the prediction results in the new data test set were low. In the present study, SVM and KNN classifiers were suggested for use as radiological diagnostic models to distinguish between parasellar cavernous hemangiomas and meningiomas.

There are several limitations in the present study. First, the sample size was relatively small and needs to be further explored. Second, different types of parasellar cavernous hemangiomas and meningiomas were not considered. Third, the differential diagnosis mainly focused on parasellar hemangiomas and meningiomas. Other parasellar tumors that are relatively easy to diagnose were not included in the study.

In conclusion, the proposed T_2_WI-based radiomics model combining SVM and KNN classifiers showed favorable predictive efficacy in the preoperative differential diagnosis between parasellar cavernous hemangiomas and meningiomas. It had more general applicability in complementing conventional imaging modalities and as an alternative to functional imaging. Moreover, the more readily available T_2_WI could provide higher detection rates and more texture features. Other imaging modalities based on T_2_WI for differentiating parasellar cavernous hemangiomas and meningiomas need to be explored.

## Supplementary Information

Supplementary Information.

## Data Availability

The datasets generated during and/or analysed during the current study are available from the corresponding author on reasonable request.
