# Supplementary material for: A radiomics-based study for differentiating parasellar cavernous hemangiomas from meningiomas
Source: Sci Rep. 2022 Sep 15;12:15509. doi: 10.1038/s41598-022-19770-9 (PMC9478116; doi:10.1038/s41598-022-19770-9)
Supplement: Supplementary file 1 — Supplementary Information. [file 41598_2022_19770_MOESM1_ESM.docx]

# Supplementary Tables

**Table S1**

Description of selected radiomic features with their associated feature group and T_1_WI filter.

| **Radiomic feature** | **Radiomic class** | **Filter** |
| --- | --- | --- |
| Skewness | firstorder | original |
| Minimum | firstorder | logarithm |
| High Gray Level Zone Emphasis | glszm | wavelet-LLH |
| Skewness | firstorder | original |
| Total Energy | firstorder | wavelet-HHL |
| Dependence Variance | gldm | gradient |
| Interquartile Range | firstorder | squareroot |
| Dependence Variance | gldm | exponential |
| Skewness Variance | firstorder | wavelet-LLL |

Label: GLDM = Gray-Level Dependence Matrix, GLSZM = Gray-Level Size Zone Matrix

**Table S2**

Description of selected radiomic features with their associated feature group and CE-T_1_WI filter.

| **Radiomic feature** | **Radiomic class** | **Filter** |
| --- | --- | --- |
| Kurtosis | firstorder | original |
| Minor Axis Length | shap | original |
| Interquartile Range | firstorder | lbp-2D |
| Kurtosis | firstorder | wavelet-HLL |
| Minimum | firstorder | wavelet-LLL |
| Interquartile Range | firstorder | original |
| Dependence Variance | gldm | wavelet-HHH |
| Minimum | firstorder | wavelet-HLL |

Label: GLDM = Gray-Level Dependence Matrix.

**Table S3**

Description of selected radiomic features with their associated feature group and ADC filter.

| **Radiomic feature** | **Radiomic class** | **Filter** |
| --- | --- | --- |
| Skewness | firstorder | logarithm |
| Skewness | firstorder | gradient |
| Large Dependence High Gray Level Emphasis | gldm | wavelet-LLH |
| Median | firstorder | logarithm |
| High Gray Level Zone Emphasis | glszm | wavelet-LLH |
| Dependence Variance | gldm | wavelet-LHH |
| Range | firstorder | wavelet-LLL |
| Maximum | firstorder | square |
| High Gray Level Zone Emphasis | glszm | wavelet-HHL |

Label: GLDM = Gray-Level Dependence Matrix, GLSZM = Gray-Level Size Zone Matrix.

**Table S4**

Description of selected radiomic features with their associated feature group and DWI filter.

| **Radiomic feature** | **Radiomic class** | **Filter** |
| --- | --- | --- |
| Joint Entropy | glcm | logarithm |
| Busyness | ngtdm | wavelet-HLL |
| Large Dependence High Gray Level Emphasis | gldm | wavelet-LHH |
| Joint Entropy | glcm | wavelet-LLL |
| Size Zone Non Uniformity | glszm | wavelet-HHL |
| Large Area High Gray Level Emphasis | glszm | wavelet-HHL |
| High Gray Level Zone Emphasis | glszm | wavelet-LLH |

Label: GLCM = Gray-Level Co-Occurrence Matrix, NGTDM = Neighbouring Gray Tone Difference Matrix, GLDM = Gray-Level Dependence Matrix, GLSZM = Gray-Level Size Zone Matrix.
